# Myokines Produced by Cultured Bovine Satellite Cells Harvested from 3- and 11-Month-Old Angus Steers

**DOI:** 10.3390/ani14050709

**Published:** 2024-02-24

**Authors:** Katie A. Shira, Brenda M. Murdoch, Kara J. Thornton, Caleb C. Reichhardt, Gabrielle M. Becker, Gwinyai E. Chibisa, Gordon K. Murdoch

**Affiliations:** 1 Animal, Veterinary, and Food Science Department, University of Idaho, Moscow, ID 83843, USA; 2 Department of Animal, Dairy and Veterinary Science, Utah State University, 4815 Old Main Hill, Logan, UT 84322, USA; 3 Department of Human Nutrition, Food and Animal Sciences, University of Hawai‘i at Manoa, 1955 East-West Rd., Honolulu, HI 96822, USA; 4 Department of Animal Sciences, Washington State University, Pullman, WA 99163, USA

**Keywords:** interleukin 6, interleukin 15, myonectin, irisin, brain-derived neurotrophic factor, Angus steer

## Abstract

**Simple Summary:**

One class of muscle growth regulators known as myokines have been vastly understudied in many livestock species, including cattle. Myokines are proteins produced by muscle that regulate muscle growth and have systemic functions that support whole-body health, as identified in many human and biomedical studies. In cattle, it is unknown how many myokines regulate growth, as very few myokines have been characterized with respect to expression, secretion, and functionality. Therefore, the expression and secretion of interleukin-6 (IL-6), interleukin-15 (IL-15), myonectin (CTRP15), fibronectin type III domain containing protein 5/irisin (FNDC5), and brain-derived neurotrophic factor (BDNF), were evaluated in this study. The results suggest and support that IL-6, IL-15, CTRP15, and BDNF are myokines secreted from bovine satellite cells and that they may contribute to the regulation of muscle stem cell activity in cattle.

**Abstract:**

The myokines interleukin 6 (IL-6), interleukin 15 (IL-15), myonectin (CTRP15), fibronectin type III domain containing protein 5/irisin (FNDC5), and brain-derived neurotrophic factor (BDNF) are associated with skeletal muscle cell proliferation, differentiation, and muscle hypertrophy in biomedical model species. This study evaluated whether these myokines are produced by cultured bovine satellite cells (BSCs) harvested from 3- and 11-month-old commercial black Angus steers and if the expression and secretion of these targets change across 0, 12, 24, and 48 h in vitro. *IL-6*, *IL-15, FNDC5*, and *BDNF* expression were greater (*p* ≤ 0.05) in the differentiated vs. undifferentiated BSCs at 0, 12, 24, and 48 h. *CTRP15* expression was greater (*p* ≤ 0.03) in the undifferentiated vs. differentiated BSCs at 24 and 48 h. IL-6 and CTRP15 protein from culture media were greater (*p* ≤ 0.04) in undifferentiated vs. differentiated BSCs at 0, 12, 24, and 48 h. BDNF protein was greater in the media of differentiated vs. undifferentiated BSCs at 0, 12, 24, and 48 h. *IL-6*, *1L-15*, *FNDC5*, and *BDNF* are expressed in association with BSC differentiation, and *CTRP15* appears to be expressed in association with BSC proliferation. This study also confirms IL-6, IL-15, CTRP15, and BDNF proteins present in media collected from primary cultures of BSCs.

## 1. Introduction

Skeletal muscle is an endocrine tissue that secretes proteins, which regulate whole-body growth and health through autocrine, paracrine, and endocrine signaling [1,2]. These proteins are often triggered for synthesis and release by muscle contractions/exercise and are classified as myokines [3]. In livestock production, there is a huge knowledge gap as to what myokine proteins are being secreted and how they may function. There are only a few cattle studies that have investigated myokines [4,5,6] relative to the almost 600 that have been documented in human studies and other biomedical models [7]. Muscle is a valuable commodity for livestock production. Therefore, understanding how muscle accretion is regulated could help enhance muscle growth and efficiency.

Primary muscle cells from humans and mice are frequently utilized to identify the expression, secretion, and function of myokines [8,9,10]. Primary muscle stem cells are also known as satellite cells, which are positioned between the basal lamina and sarcolemma of the muscle fiber [11]. During postnatal muscle growth, the heterogenous satellite cell pool works to maintain the quiescent satellite cell pool as well as actively contribute to myonuclear accretion. This then promotes hypertrophic muscle growth through proliferation and fusion of cells with the existing muscle fibers [12]. In cattle, these stem cells are commonly referred to as bovine satellite cells (BSCs) [13,14,15]. BSCs were used in this study to confirm myokine targets in vitro and if they were produced during cellular proliferation or differentiation.

The myokine targets evaluated in this study were interleukin 6 (IL-6), interleukin 15 (IL-15), myonectin (CTRP15), fibronectin type III domain containing protein 5 (FNDC5), and brain-derived neurotrophic factor (BDNF). Each of these proteins is released in an exercise-induced manner from the skeletal muscle of rodents and humans [16,17]. Although exercise is not used as a strategy to induce skeletal muscle growth in production livestock animals, muscle contraction plays an important role in animal health and meat quality, and contraction could lead to the expression and secretion of these myokines. In addition to identifying these proteins in cattle, they also need to be understood in regard to how they contribute to meat quality, as this is an important factor in the production of meat-producing livestock. It is unknown how these proteins directly affect meat quality in livestock, but some of these targets have been proposed to be evaluated due to their influence in other species [18] and should be evaluated in the actual animal. Therefore, it is important to take the initial step and evaluate whether each of these potential myokines is expressed and secreted by bovine skeletal muscle in vivo to determine their potential role in regulating growth.

IL-6 is most associated with being an inflammatory cytokine; it has been shown to aid in skeletal muscle regeneration and repair [19,20,21]. Recently, IL-6 was demonstrated to be an energy-sensing myokine, with its secretion leading to the translocation of the glucose transporter type 4 (GLUT4) to the cell surface, which enables glucose uptake into muscle cells [22]. IL-15 is also known for its ability to aid in murine skeletal muscle repair and regeneration, as previously demonstrated in an overexpression study [23]. It is considered an anabolic growth factor since it promotes the deposition of the myosin heavy chain in differentiated muscle cells [24,25]. Little is known about the potential role of CTRP15 in bovine muscle. *CTRP15* expression has been shown to decrease with age, as identified in mouse models, and is highly expressed in murine skeletal muscle compared to other tissues [26]. These roles are important to understand in bovine muscle as they may influence growth rates, maturation, and longevity in livestock. The *FNDC5* gene encodes for the FNDC5 protein, and through proteolytic cleavage and glycosylation, a new protein/myokine referred to as irisin is produced [27]. *FNDC5* has been evaluated in a study using Charolais × German Holstein bulls, where its expression was identified in skeletal muscle, but there was an inability to detect muscle and plasma irisin [5]. Irisin regulates glucose uptake by muscle cells by stimulating the translocation of GLUT4 to the cell surface in a similar manner to IL-6 and insulin [28]. BDNF is very important for maintaining the neural system and is expressed by many tissues [29,30], including skeletal muscle, as identified in mouse models [31]. In C2C12 mouse cells, *BDNF’s* expression was verified during proliferation and decreased under conditions of myogenic differentiation [32]. Each of these myokines has very important roles in tissue maintenance, growth, and metabolism, as identified in biomedical models, but they have yet to be fully identified or characterized in cattle and other livestock species. Through the previously described functions of these proteins, it is possible that they could be powerful skeletal muscle growth regulators in cattle and could also contribute to animal health.

The objective of this study was to evaluate the expression and secretion patterns of IL-6, IL-15, CTRP15, FNDC5, and BDNF in undifferentiated and differentiated primary BSCs harvested from young (3-month-old (BSC3)) and older (11-month-old (BSC11)) steers. In many stem cell types, including muscle stem cells, changes with age correlate with decreased capacity for activation and maturation [33]. It is unknown whether there are myokine-specific age differences in cattle, and using these age categories provides cattle from two different developmental stages that are production-relevant. Additionally, at these stages, the BSCs are still active, and as mammals age, the cells become quiescent and can be more challenging to culture [33,34].

## 2. Materials and Methods

### 2.1. BSC Isolation

Commercially raised Angus-based steers that were 3 (*n* = 3) and 11 months old (*n* = 3) were humanely harvested following preapproved Institutional Animal Care and Use Committee guidelines at the University of Idaho (protocol # 017090) and Utah State University (protocol # 10216), respectively. Selected steers did not receive FDA hormonal anabolic implants in their lives prior to harvest. The immunization protocols for steers were unknown, but steers were considered healthy for harvest. After exsanguination, the semimembranosus muscle was obtained and placed in 4 °C phosphate-buffered saline (PBS) with 3x antibiotic–antimycotic (ABAM) (Life Technologies Inc., Grand Island, NY, USA). Transportation of the muscle from the harvest facility to the lab, which occurred right after acquisition, took five minutes. Once at the lab, the BSCs were isolated from the semimembranosus muscle as previously described [35], and all reagents and media were phenol red-free.

### 2.2. BSC Culture

A culture of BSCs was developed, as previously described [36]. Cells were seeded in wells that were 4 cm^2^ across 12-well tissue culture plates at a density of 2 g/cm^2^. BSCs were resurrected and seeded in Dulbecco’s Modified Eagle Medium (DMEM) (Corning, Manassas, VA, USA), 10% fetal bovine serum (FBS) (HyClone Laboratories, Logan, UT, USA), growth media containing 2x L-glutamine (Life Technologies, Grand Island, NY, USA), and 1x ABAM. Growth factor-reduced Corning^®^ Matrigel Matrix (Corning, Discovery Labware Inc., Bedford, MA, USA), which was diluted 1:50 (*v*/*v*), was used to pre-coat wells as previously described [35]. Three biological, technical, and experimental replicates were used. Biological replicates were the steers from each age group (*n* = 3); technical replicates were cells plated across 3 wells for each experiment; and the experiment was replicated three times.

### 2.3. Treatments

Cells were first grown to 70% confluency before changing the medium a final time. The old growth media was discarded, and cells were washed twice with warm PBS (1 mL) containing 1x ABAM. Two separate experiments were run, one on cells induced into differentiation and one on cells that were not induced into differentiation and were classified as undifferentiated cells. Undifferentiated cells received normal growth media, and cells induced into differentiation received differentiation media. The differentiation media contained DMEM, 3% horse serum (Life Technologies, Grand Island, NY, USA), and 1.5% bovine serum albumin–linoleic acid (BSA–LA) (Sigma-Aldrich, Burlington, MA, USA). This change of media is what initiated experimental time 0.

### 2.4. Sample Collection

After media treatments were added, 0 h plates were imaged. Media and RNA samples were collected immediately after imaging the cells. The media was aspirated off the cells, placed in microcentrifuge tubes, and placed directly in a −80 °C freezer. Tissue culture plates were then placed directly on ice, and cells were washed twice with cold PBS containing 1x ABAM to remove any remnants of media. After cells were washed, lysis buffer from Absolutely RNA Miniprep Kits (Agilent Technologies, Cedar Creek, TX, USA) was prepared according to the manufacturer’s instructions and placed on cells to initiate cell lysis. Once lysis buffer was administered, cells were further collected using a sterile cell scraper. After mechanically lyzing the cells, the supernatant was aspirated, placed in microcentrifuge tubes, and immediately placed in a −80 °C freezer until the remainder of the isolation process was completed. This was performed for both treatments with all cells. The other plates were placed in the incubator prior to sample collection at 12, 24, and 48 h, and subsequently, the same collection process as previously described was utilized. At each timepoint, each well of cells was imaged three times to capture a representation of cell confluency, and sample collection was performed in a sterile laminar hood.

### 2.5. Cell Confluency

**Figure 1:**
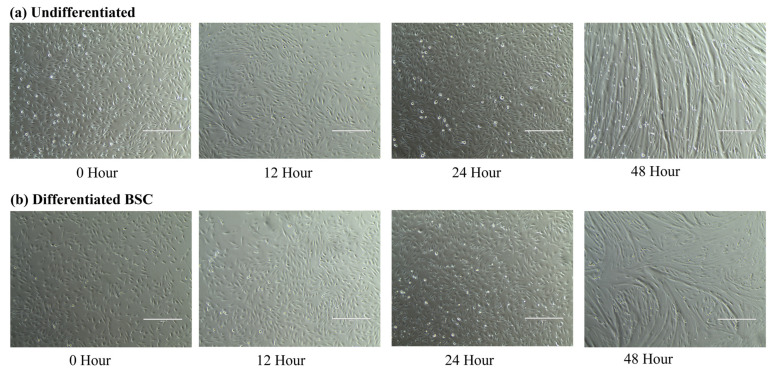
Cell growth from undifferentiated and differentiated cell treatments. Cells were visualized at 100× across 0, 12, 24, and 48 h. (**a**) Undifferentiated BSCs. (**b**) Differentiated BSCs.

At 0 h, cells were at 70% confluency, as previously mentioned. At 24 and 48 h, cells visibly began to fuse and become more organized (Figure 1). Substantial fusion was observed in both treatments, classified as undifferentiated and differentiated at 48 h. Undifferentiated cells undergo fusion and differentiation as a natural result of high cell confluency.

### 2.6. Protein Analysis

Culture media was removed from each well and placed into 2 mL microcentrifuge tubes. The media was stored at −80 °C until protein analysis, with freeze-thaw of samples kept to a minimum. The remaining cells were collected for RNA isolation. For protein analysis, media samples were first thawed on ice. After thawing, each sample was analyzed for bovine interleukin 15 (IL-15) (catalogue #: MBS2609359), interleukin 6 (IL-6) (MBS2023586), brain-derived neurotrophic factor (BDNF) (MBS285453), and erythroferrone/myonectin (CTRP15) (MBS7263680) using commercial enzyme-linked immunosorbent assay (ELISA) kits (MyBiosource Inc.; San Diego, CA, USA) according to the manufacturer’s instructions. Assays for IL-6, IL-15, and BDNF had an intra-assay coefficient of variation (CV) of <8% and an inter-assay CV of <12%, and ERFE had an inter-assay CV of <10% from the same lot and 12% from different lots. Clean unconditioned media was also analyzed in each ELISA assay for both the undifferentiated and differentiated growth media to identify whether any of these proteins were detectable in clean unconditioned media (Table 1). The data in Table 1 were not used to calculate any values in the dataset, but it is a reference to show the natural levels of each myokine protein present in the growth media used for this study. The protein in this study was not normalized to total protein, but the volume of the media used for protein extraction was standardized within each assay. The data were reported as protein quantified per volume of media.

### 2.7. RNA Isolation, Quantification, and cDNA Synthesis

Absolutely RNA Miniprep Kits (Agilent Technologies, Cedar Creek, TX, USA) were used for RNA isolation from cells as previously described [35,37]. After RNA isolation, quantification took place using a NanoDrop™ One instrument (ThermoFisher Scientific Inc., Waltham, MA, USA). Samples with an acceptable quantity of RNA were selected for analysis, diluted to a 20 ng/μL concentration, and used for cDNA synthesis. All samples were converted into cDNA using a high-capacity cDNA reverse transcription kit (Applied Biosystem, Foster City, CA, USA).

### 2.8. Quantitative Real-Time PCR

Primers and probes for real-time quantitative PCR (Table 2) were designed to be TaqMan™ MGB and QSY Probes (ThermoFisher Scientific Inc., Waltham, MA, USA) using Primer Express™ 3 v. 3.0.1 (Life Technologies, Corp., Waltham, MA, USA). Primers were acquired through Integrated DNA Technologies Inc. (Coralville, IA, USA). For gene expression analysis, primers were brought to a 40 μM concentration and probes to a 10 μM concentration. TaqMan Fast Advanced Master Mix (Applied Biosystems, USA) was used with all samples. The relative mRNA abundance of each gene of interest was assessed with a Life Technologies ViiA7 Real-Time PCR System (Applied Biosystems, Foster City, CA, USA). Reactions were 10 μL fast reactions run for 50 cycles as comparative CT experiments. The run parameters for these assays were as follows: hold stage: 50 °C for 2 min, and 95 °C for 20 s; PCR stage: 95 °C for 1 s, and 60 °C for 20 s. This was for all targets except BDNF, which was run at 58 °C for 20 s during the PCR stage. A lower annealing temperature was optimal based on the primer design. All samples were analyzed in duplicate for mRNA expression for each target. Prior to statistical analysis of the mRNA data, the data were normalized utilizing ribosomal 18S as our internal reference gene via the delta Ct (ΔCt) method to yield relative mRNA data for each gene of interest, and the data were presented at 2^−ΔCT^. This housekeeping gene has been found to be sufficient and in range for bovine skeletal muscle [38] and has been used by others as the internal reference for bovine skeletal muscle research [39,40].

### 2.9. Statistical Analysis

The GLIMMIX procedure in SAS^®^ (version 9.4; SAS Institute Inc., Cary, NC, USA) was used for all analyses. No transformations were used with the data, and distributions were specified and accounted for in the model [41]. The fixed effects were age and time, and the response variable was the myokine of interest being tested. Each animal was the experimental unit, and there were three experimental replicates with three technical replicates within. A regression was completed for the fixed effects of age, time, and age × time. Age accounts for the age of the animal that cells were harvested from. Significance was declared at *p* ≤ 0.05, and trends were set at *p* ≤ 0.10. Contrasts were used to identify whether there were differences between 0 h and the other time points (12, 24, and 48 h). Further, a Least Squares means analysis (LS Means) was incorporated to compare group means of BSC3 vs. BSC11 by h (age × time) and sliced by h to identify differences between the age groups within an h with Tukey–Kramer adjustments. The same was performed for the undifferentiated vs. differentiated BSCs by hour (cell treatment × time) sliced by hour to again identify within an hour if there was a difference between the differentiated and undifferentiated BSCs with Tukey-Kramer adjustments. The data were visualized using RStudio v. 4.1.2 using ggplot2 [42]. All the data represented were within two standard deviations of the mean. Whiskers on the boxplots represent 1.5 times the interquartile range.

## 3. Results

*IL-6*, *IL-15*, *CTRP15*, *FNDC5*, and *BDNF* mRNA expression levels and IL-6, IL-15, CTRP15, and BDNF protein secretion levels were evaluated using primary bovine muscle cells. The cultured cells were used to quantify mRNA expression. The medium that the cells were cultured in was used to quantify each myokine protein of interest.

### 3.1. IL-6 mRNA Analysis

**Figure 2:**
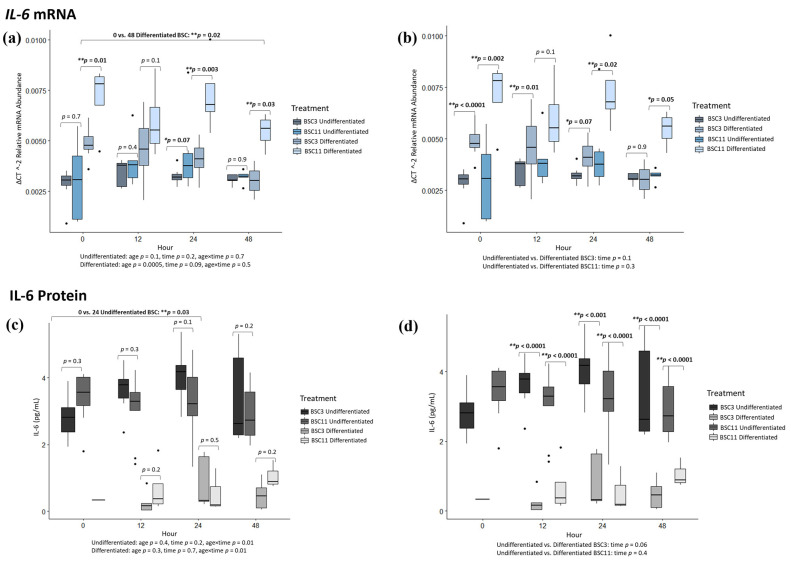
Average IL-6 mRNA expression and protein secretion. The fixed effect results are listed at the bottom of each figure. (**a**) mRNA of undifferentiated and differentiated BSC3 vs. BSC11; (**b**) mRNA of undifferentiated vs. differentiated BSC3 and undifferentiated vs. differentiated BSC11; (**c**) proteins of undifferentiated and differentiated BSC3 vs. BSC11; (**d**) proteins of undifferentiated vs. differentiated BSC3 and undifferentiated vs. differentiated BSC11. The mRNA is in relative abundance and normalized to 18S ribosomal RNA. Whiskers represent 1.5 times the interquartile range, and * represents a trend; ** represents significance. Figures report the means of three repeated measures, and BSC3 came from three-month-old steers (*n* = 3) and BSC11 came from 11-month-old steers (*n* = 3).

*IL-6* mRNA in undifferentiated BSC3 vs. BSC11 had no differences (*p* ≥ 0.4) for 0, 12, and 48 h, but there was a difference (*p* = 0.04) at 24 h (Figure 2a). The undifferentiated BSC3 had slightly less (*p* = 0.04) mRNA expression than BSC11 at this timepoint. The combined undifferentiated BSC3 and BSC11 data had trends (*p* ≤ 0.07) for 0 vs. 12 and 0 vs. 24 h, but no difference for 0 vs. 48 h (Figure 2a). There were no differences (*p* ≥ 0.1) for fixed effects of age, time, or age × time. Similarly, for the differentiated BSC3 vs. BSC11, there were differences (*p* ≤ 0.03) for 0, 24, and 48 h, but no significant difference (*p* = 0.1) for 12 h (Figure 2a). At 0, 24, and 48 h, differentiated BSC11 expressed higher *IL-6* than differentiated BSC3. The combined differentiated data had no differences (*p* ≥ 0.2) for 0 vs. 12 and 0 vs. 24 h, but there was a difference (*p* = 0.02) comparing 0 vs. 48 h (Figure 2a). From 0 to 48 h, expression levels decreased for both differentiated BSC3 and BSC11. There was a difference for the fixed effect of age (*p* = 0.0005), a trend for time (*p* = 0.09), and no difference for age × time (*p* = 0.5). The differentiated BSC11 had much higher (*p* = 0.005) expression than BSC3 at most time points.

To further elucidate whether there were differences within an age group between the treatments, comparisons were made for undifferentiated vs. differentiated BSC3. There were differences (*p* ≤ 0.01) for 0 and 12 h, a trend (*p* = 0.07) at 24 h, and no difference (*p* = 0.9) at 48 h (Figure 2b). At 0, 12, and 24 h, differentiated BSC3 expresses higher levels of *IL-6* than undifferentiated BSC3. The combined BSC3 data showed no difference (*p* ≥ 0.3) for 0 vs. 12 and 0 vs. 24 h, but there was a trend (*p* = 0.08) for 0 vs. 48 h. The expression of *IL-6* decreases from 0 to 48 h, especially in the differentiated BSC3. There was no significant difference for the overall fixed effect of time (*p* = 0.1). Undifferentiated vs. differentiated BSC11 had observable differences (*p* ≤ 0.05) at 0, 24, and 48 h and no significant difference (*p* = 0.1) at 12 h (Figure 2b). Differentiated BSC11 had higher *IL-6* expression than undifferentiated BSC11 at hour, where differences and trends were observed. The combined BSC11 data did not have differences for 0 vs. 12 (*p* = 0.7), 0 vs. 24 (*p* = 0.2), and 0 vs. 48 h (*p* = 0.6), or for the overall fixed effect of time (*p* = 0.3).

These comparisons revealed that *IL-6* expression appears to be upregulated when cells are induced into differentiation but not in normally proliferating cells. Additionally, when differentiation is induced, there is an age-related difference where cells from older steers (BSC11) express more *IL-6* than cells from younger steers (BSC3). BSC11 expressed the highest levels of IL-6 at 0 h, and with time, the expression slowly decreased with a statistical difference between 0 and 48 h, as mentioned previously. These results suggest that *IL-6* could be associated with myogenic differentiation in BSCs.

### 3.2. IL-6 Protein Analysis

IL-6 had no observed differences (*p* ≥ 0.1) when comparing BSC3 vs. BSC11 within an hour at 0, 12, 24, or 48 h (Figure 2c). There were also no differences (*p* ≥ 0.3) when combining the undifferentiated BSC3 and BSC11 for 0 vs. 12 or 0 vs. 48 h, but there was a difference (*p* = 0.03) comparing 0 vs. 24 h. There was an increase in protein levels at 24 h in comparison to 0 h in the undifferentiated BSCs. As for the overall fixed effects, there were no differences (*p* ≥ 0.2) for the fixed effects of age or hour, but there was a difference (*p* = 0.01) for age × time. Differentiated BSC3 vs. BSC11 had no differences (*p* ≥ 0.2) at 12, 24, and 48 h (Figure 2c). The 0 h comparison was not possible as IL-6 was undetected at this time point. This also made the comparison of 0 vs. 12, 0 vs. 24, and 0 vs. 48 h not possible when looking at the differentiated data as a whole. The overall fixed effects from the differentiated BSCs had no differences (*p* ≥ 0.3) for fixed effects age, time, or age × time.

Undifferentiated vs. differentiated BSC3 compared within an hour had differences (*p* ≤ 0.0001) at 12, 24, and 48 h (Figure 2d). Undifferentiated BSC3 had higher levels of IL-6 protein in the culture media at each of these times compared to differentiated BSC3. With insufficient data at 0 h, the comparison of 0 to each hour of interest was not made. There was a trend for the fixed effect of time (*p* = 0.06). Undifferentiated vs. differentiated BSC11 compared within an hour had differences (*p* < 0.0001) for 12, 24, and 48 h (Figure 2d). The differentiated BSCs had substantially less IL-6 in the medium than the undifferentiated BSCs. There was no comparison made with the 0 h timepoint as there was insufficient data for this timepoint, as previously mentioned. As for the fixed effect of time, there was also no difference observed (*p* = 0.4).

The protein results of IL-6 showed that BSCs secrete more IL-6 into the media when cells are not induced to differentiate. The mRNA results suggest that the BSCs would secrete more IL-6 from the differentiated BSC11, but that was not observed, and as we know, there is often both a timing and regulatory difference between mRNA and protein detection. Regardless, it was surprising to find very low levels of protein in the differentiated media samples. This study did not evaluate the receptors for any of the proteins on or in the cells, but there is a possibility that the cells bind IL-6 that was present in the media in both the undifferentiated and differentiated treatments. This would be one reason why very low levels are detected in the media. The authors reported difficulty detecting the IL-6 protein from the differentiated BSC treatment specifically, and there is a possibility that the horse serum in the differentiation media could be interacting with the ELISA. Overall, these data indicate that IL-6 was detected in BSCs both through gene expression and in the media, confirming it as a myokine from BSCs. It would be valuable to conduct a follow-up study to evaluate IL-6 receptors on BSCs and test other ELISA assays to further validate these results and see if the mRNA and protein results would agree with additional ELISA tests.

### 3.3. IL-15 mRNA Analysis

**Figure 3:**
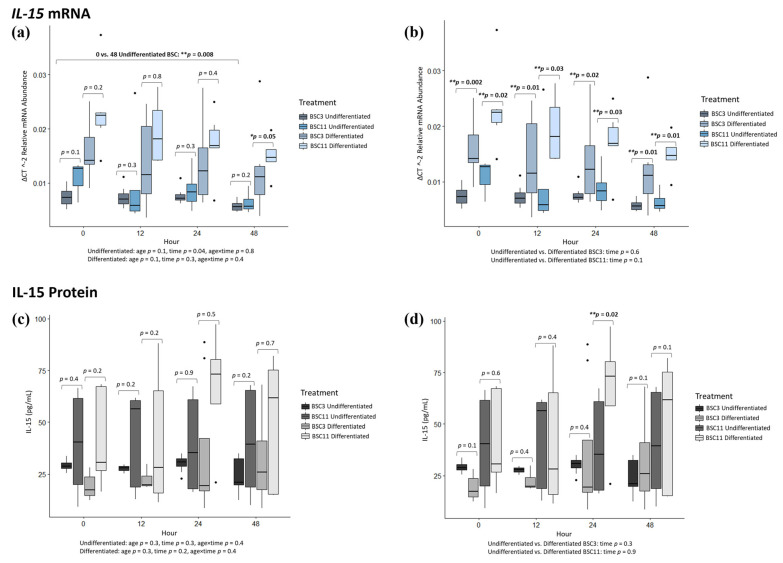
Average IL-15 mRNA expression and protein secretion. The fixed effect results are listed at the bottom of each figure. (**a**) mRNA of undifferentiated and differentiated BSC3 vs. BSC11; (**b**) mRNA of undifferentiated vs. differentiated BSC3 and undifferentiated vs. differentiated BSC11; (**c**) proteins of undifferentiated and differentiated BSC3 vs. BSC11; (**d**) proteins of undifferentiated vs. differentiated BSC3 and undifferentiated vs. differentiated BSC11. The mRNA is in relative abundance and normalized to 18S ribosomal RNA. Whiskers represent 1.5 times the interquartile range, and * represents a trend; ** represents significance. Figures report the means of three repeated measures; BSC3 came from three-month-old steers (*n* = 3); and BSC11 came from 11-month-old steers (*n* = 3).

*IL-15* expression analysis showed that undifferentiated BSC3 vs. BSC11 had no observed differences (*p* ≥ 0.1) when comparing within an hour for 0, 12, 24, and 48 h between BSC3 and BSC11 (Figure 3a). Looking at the combined BSC3 and BSC11 undifferentiated data, there were no differences (*p* ≥ 0.3) for 0 vs. 12 and 0 vs. 24 h, but there was a difference (*p* = 0.008) for 0 vs. 48 h (Figure 3a). From 0 to 48 h, there was a decline in mRNA expression of *IL-15*, especially in the undifferentiated BSC11. For the overall fixed effects of the differentiated data, there was a difference for time but no difference (*p* ≥ 0.1) for age or age × time. Over time, it appears that the undifferentiated mRNA for *IL-15* decreases (*p* = 0.04) by hour. Differentiated BSC3 vs. BSC11 had no difference (*p* ≥ 0.2) for 0, 12, and 24 h, but there was a difference at 48 h (*p* = 0.05) (Figure 3a), which is due to differentiated BSC3 having lower *IL-15* expression than BSC11. As for the combined differentiated data, there was a trend for the comparison of 0 vs. 12 h (*p* = 0.09), but no difference (*p* ≥ 0.3) for 0 vs. 24 and 0 vs. 48 h (Figure 3a). There were no differences (*p* ≥ 0.2) for the fixed effects of age, time, and age × time.

Undifferentiated vs. differentiated BSC3 had differences (*p* ≤ 0.02) for 0, 12, 24, and 48 h (Figure 3b). The combined BSC3 data showed no difference (*p* ≥ 0.2) for 0 vs. 12, 0 vs. 24, and 0 vs. 48 h (Figure 3b). There were also no differences for the fixed effect of time (*p* = 0.6). Undifferentiated vs. differentiated BSC11 had differences (*p* ≤ 0.03) at 0, 12, 24, and 48 h (Figure 3b). Differentiated BSC11 had higher *IL-15* mRNA expression at each of these times than undifferentiated BSC11. The BSC11 data showed no difference (*p* ≥ 0.2) for 0 vs. 12 and 0 vs. 24 h (Figure 3b). There was a difference (*p* = 0.03) for 0 vs. 48 h, where 0 h has higher mRNA expression than the 48 h timepoint. There was no difference (*p* = 0.1) for the fixed effect of time.

These results show that when differentiation is induced, there is an increase in *IL-15* expression, regardless of the age of the animal the BSCs were harvested from. There were no apparent or statistical differences within the undifferentiated treatment of cells.

### 3.4. IL-15 Protein Analysis

Undifferentiated BSC3 vs. BSC11 had no differences (*p* ≥ 0.2) for 0, 12, 24, and 48 h (Figure 3c). There was a large variation in undifferentiated BSC11, which was surprising, and this contributed to there being no observed differences across all tests. Combining BSC3 and BSC11 undifferentiated data, there were no differences (*p* ≥ 0.1) for 0 vs. 12, 0 vs. 24, and 0 vs. 48 h (Figure 3c). Additionally, there were no differences (*p* ≥ 0.3) for fixed effects of age, time, or age × time. Differentiated BSC3 vs. BSC11 had no difference (*p* ≥ 0.2) for 0, 12, 24, and 48 h (Figure 3c). There were no differences (*p* ≥ 0.3) for 0 vs. 12, 0 vs. 24, and 0 vs. 48 h (Figure 3c), or for the fixed effects of age, time, or age × time.

Undifferentiated vs. differentiated BSC3 had no differences (*p* ≥ 0.1) for 0, 12, 24, and 48 h (Figure 3d). There were also no differences (*p* ≥ 0.2) for the combined undifferentiated and differentiated BSC3 data for 0 vs. 12, 0 vs. 24, and 0 vs. 48 h (Figure 3d), or for the fixed effect of time. Undifferentiated vs. differentiated BSC11 had no differences (*p* ≥ 0.1) for 0, 12, and 48 h (Figure 3d). There was a difference (*p* = 0.02) at 24 h, where IL-15 protein in the media was higher in differentiated BSC11 compared to the undifferentiated BSC11. Combined BSC11 data also had no difference (*p* ≥ 0.5) for 0 vs. 12, 0 vs. 24, and 0 vs. 48 h (Figure 3d) or for a fixed effect of time.

From these results, it was determined that there are no statistical differences across all of the protein data except for at 24 h, where differentiated BSC11 was elevated in secretion. It is important to note that IL-15 protein was detected in the treatment at a greater quantity than the unconditioned media in Table 1, especially from the differentiation treatments. The authors are confident that IL-15 proteins were secreted from the BSCs, confirming it as a myokine. There was variation in the protein results of the differentiated BSCs, which is why the authors believe there were no differences observed. The authors are still confident in the result that these proteins are secreted from BSCs overall, confirming them as myokines.

### 3.5. CTRP15 mRNA Analysis

**Figure 4:**
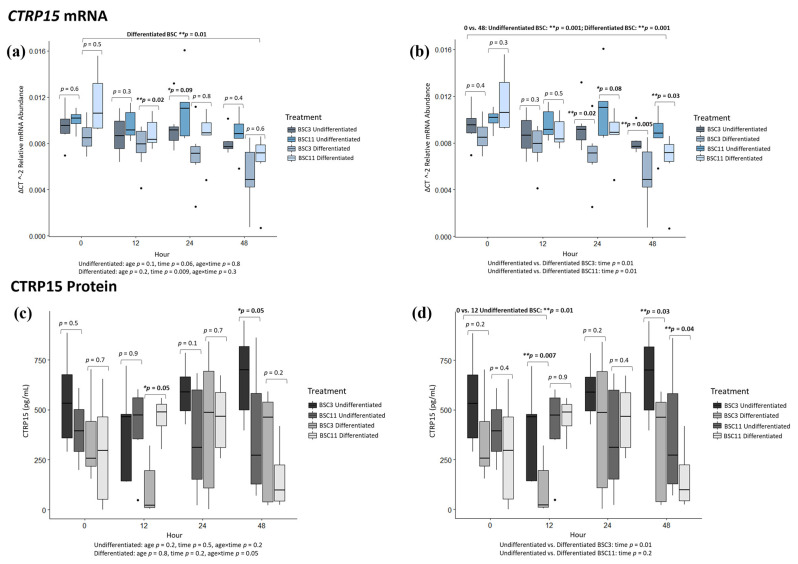
Average CTRP15 mRNA expression and protein secretion. The fixed effect results are listed at the bottom of each figure. (**a**) mRNA of undifferentiated and differentiated BSC3 vs. BSC11; (**b**) mRNA of undifferentiated vs. differentiated BSC3 and undifferentiated vs. differentiated BSC11; (**c**) proteins of undifferentiated and differentiated BSC3 vs. BSC11; (**d**) proteins of undifferentiated vs. differentiated BSC3 and undifferentiated vs. differentiated BSC11. The mRNA is in relative abundance and normalized to 18S ribosomal RNA. Whiskers represent 1.5 times the interquartile range, and * represents a trend; ** represents significance. Figures report the means of three repeated measures; BSC3 came from three-month-old steers (*n* = 3); and BSC11 came from 11-month-old steers (*n* = 3).

Undifferentiated BSC3 vs. BSC11 had no difference (*p* ≥ 0.3) for 0, 12, and 48 h, but there was a trend at 24 h where *CTRP15* from undifferentiated BSC11 appears elevated (*p* = 0.09) compared to BSC3 (Figure 4a). The combined undifferentiated BSC data also had no differences (*p* ≥ 0.4) for 0 vs. 12 and 0 vs. 24 h, but there was a trend (*p* = 0.05) comparing 0 vs. 48 h (Figure 4a). Undifferentiated BSC3 expression dropped between 0 and 48 h. There were no differences for the fixed effects (*p* ≥ 0.1) of age or for age × time, but there was a trend (*p* = 0.06) for the fixed effect of time. Differentiated BSC3 vs. BSC11 had no difference (*p* ≥ 0.5) for 0, 24, and 48 h, but there was a difference (*p* = 0.02) at 12 h (Figure 4a). The differentiated BSC3 expressed less *CTRP15* mRNA than the BSC11 did at this time. Combining the differentiated BSC3 and BSC11, there were no differences (*p* ≥ 0.1) for the comparisons of 0 vs. 12 and 0 vs. 24 h, but there was a difference (*p* = 0.01) for 0 vs. 48 h (Figure 4a). This significance can still be attributed to the decrease in expression from 0 to 48 h. There were no differences (*p* ≥ 0.2) for fixed effects of age (*p* = 0.2) and age × time (*p* = 0.3), but there was a difference (*p* = 0.009) for time, as over the time points gene expression decreased for the differentiated BSCs.

Undifferentiated vs. differentiated BSC3 had no differences (*p* ≥ 0.3) in *CTRP15* expression for 0 and 12 h, but there were differences (*p* ≤ 0.02) at 24 and 48 h (Figure 4b). At both 24 and 48 h, *CTRP15* expression is higher in undifferentiated BSC3 than in differentiated BSC3. The combined BSC3 data had no difference (*p* ≥ 0.2) for 0 vs. 12 and 0 vs. 24 h, but there was a difference (*p* = 0.001) for 0 vs. 48 h (Figure 4b). The *CTRP15* mRNA expression was lower at 48 h than at 0 h. There was a difference (*p* = 0.01) for the fixed effect of time. Undifferentiated vs. differentiated BSC11 had no difference (*p* ≥ 0.3) for 0 and 12 h, but there was a trend (*p* = 0.08) at 24 h and a difference (*p* = 0.03) at 48 h (Figure 4b). The undifferentiated BSC11 expression was elevated at 24 h in comparison to the differentiated BSC11, and it was even higher at 48 h, which led to the significant difference. Combining the BSC11 data, there were no differences (*p* ≥ 0.2) for 0 vs. 12 and 0 vs. 24 h, but there was a difference (*p* = 0.001) for 0 vs. 48 h (Figure 4b). There was a difference for the fixed effect of time (*p* = 0.01) as *CTRP15* from BSC11 decreased in expression across the time points tested.

These results overall indicate that there is a decrease in *CTRP15* expression across time points for both the undifferentiated and differentiated BSC treatments. Additionally, at 24 and 48 h, the undifferentiated BSCs had elevated expression compared to the differentiated BSC. From these expression results, we would associate *CTRP15* with proliferation rather than differentiation, as seen in the other targets evaluated in this study.

### 3.6. CTRP15 Protein Analysis

There were very few differences in the protein results, but CTRP15 was detectable. For the comparison of undifferentiated BSC3 vs. BSC11, there was no difference (*p* ≥ 0.5) for 0, 12, and 24 h. The only difference (*p* = 0.05) was observed at 48 h (Figure 4c), and at this time, the undifferentiated BSC3 culture media had higher CTRP15 protein than the undifferentiated BSC11 media. There were no differences (*p* ≥ 0.2) for combined undifferentiated BSC3 and BSC11 for 0 vs. 12, 0 vs. 24, and 0 vs. 48 h, and fixed effects of age, time, and age × time. Differentiated BSC3 vs. BSC11 had no difference (*p* ≥ 0.2) for 0, 24, and 48 h (Figure 4c). There were also no differences (*p* ≥ 0.2) when combining the data for 0 vs. 12, 0 vs. 24, and 0 vs. 48 h, or for age and time, but there was a trend (*p* = 0.05) for age × time.

Undifferentiated BSC3 vs. differentiated BSC3 had differences (*p* ≤ 0.03) for 12 and 48 h, but no difference (*p* ≥ 0.2) at 0 and 24 h (Figure 4d). The combined BSC3 data showed a difference (*p* = 0.01) for 0 vs. 12 h (Figure 4d), where 0 h had higher CTRP15 protein than at 12 h for both cell types. There was no difference (*p* ≥ 0.2) for 0 vs. 24 and 0 vs. 48 h, and for the fixed effect of time. Undifferentiated BSC11 vs. differentiated BSC11 had no difference (*p* ≥ 0.4) for 0, 12, and 24 h, but there was a difference (*p* = 0.04) at 48 h (Figure 4d). At 48 h, the undifferentiated BSC11 had higher levels (and more variable levels) of CTRP15 in the media than the differentiated BSC11. For the combined BSC11 data, culture media did not have a difference (*p* ≥ 0.2) for the comparisons of 0 vs. 12, 0 vs. 24, and 0 vs. 48 h, or the fixed effect of time.

As seen in Figure 4c, where differences in the protein did occur, the undifferentiated BSCs secreted more protein than the BSCs induced into differentiation. The protein results do align more with the mRNA here than with some of the other targets. *CTRP15* is higher in undifferentiated BSCs and appears to be downregulated in differentiated BSCs at certain time points. CTRP15 protein started at a similar level in treatments as in the unconditioned media (Table 1). Even though the values start similar, there are fluctuations in the protein assessed from the treatments. The protein goes down initially in some of the age and treatment groups, and then it goes back up. The reason that protein levels could be decreasing would again possibly be attributed to proteins being bound by receptors, but that was not tested. The decrease and increase again do show that these proteins are produced by the BSCs, confirming them as myokines.

### 3.7. FNDC5 mRNA Analysis

**Figure 5:**
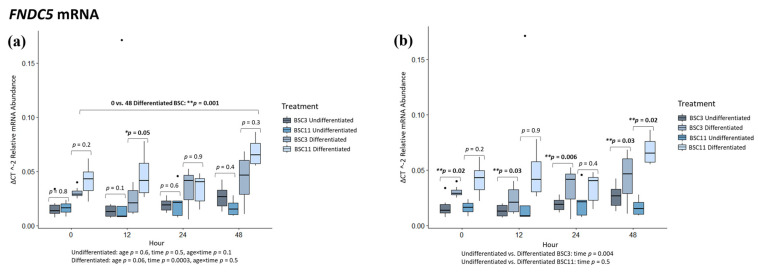
Average *FNDC5* mRNA expression. The fixed effect results are listed at the bottom of each figure. (**a**) mRNA of undifferentiated and differentiated BSC3 vs. BSC11; (**b**) mRNA of undifferentiated vs. differentiated BSC3 and undifferentiated vs. differentiated BSC11. The mRNA is in relative abundance and normalized to 18S ribosomal RNA. Whiskers represent 1.5 times the interquartile range, and * represents a trend; ** represents significance. Figures report the means of three repeated measures; BSC3 came from three-month-old steers (*n* = 3); BSC11 came from 11-month-old steers (*n* = 3).

Only the relative abundance of *FNDC5* mRNA was measured in this study. The detection of irisin protein cleaved from FNDC5 protein has been challenging, as outlined by others [43,44,45], and could not be quantified in this study as there is no accurate method for measuring it. Undifferentiated BSC3 vs. BSC11 had no difference (*p* ≥ 0.1) for 0, 12, 24, and 48 h (Figure 5a); for the comparisons of 0 vs. 12, 0 vs. 24, and 0 vs. 48 h (Figure 5a); and for the fixed effects of age, time, or age × time. Differentiated BSC3 vs. BSC11 had no differences (*p* ≥ 0.2) at 0, 24, and 48 h, but there was a difference (*p* = 0.05) at 12 h (Figure 5a), where the differentiated BSC11 expressed more *FNDC5* than the differentiated BSC3. There were no differences (*p* ≥ 0.5) for 0 vs. 12 and 0 vs. 24 h, but there was a difference (*p* = 0.001) for 0 vs. 48 h (Figure 5a). The expression increased for both differentiated BSC3 and BSC11 from 0 to 48 h. There was a difference (*p* = 0.0003) for the fixed effect of time, a trend (*p* = 0.06) for age, and no difference (*p* = 0.5) for age × time. The differentiated BSC11 expressed higher levels of *FNDC5* than the differentiated BSC3, and the expression appeared to increase over time.

Undifferentiated vs. differentiated BSC3 had differences (*p* ≤ 0.03) for 0, 12, 24, and 48 h (Figure 5b). At each of these times, differentiated BSC3 expressed higher mRNA than undifferentiated BSC3. The combined BSC3 data had no differences (*p* ≥ 0.2) for 0 vs. 12 and 0 vs. 24 h, but there was a difference (*p* = 0.02) for 0 vs. 48 h (Figure 5b). There was a difference (*p* = 0.004) for the fixed effect of time. Across each of the time points, mRNA expression increased, especially in samples of the differentiated BSC3. The undifferentiated BSC11 vs. differentiated BSC11 had no differences (*p* ≥ 0.2) for 0, 12, and 24 h. There was a difference (*p* = 0.02) between the differentiated and undifferentiated BSC11 at 48 h (Figure 5b), where the differentiated BSCs expressed a much higher level of *FNDC5* than the undifferentiated BSC. The combined BSC11 data showed no difference (*p* ≥ 0.2) for 0 vs. 12, 0 vs. 24, and 0 vs. 48 h (Figure 5b), or for a fixed effect of time.

There was significantly more *FNDC5* expression in the differentiated samples compared to the undifferentiated BSC. The intent here was to measure the myokine irisin, and this gene encodes for irisin. This only shows us that the gene is expressed and that there are some differences in its expression between treatments, but this does not provide an indication as to how much irisin is produced or if there would also be differences in its production.

### 3.8. BDNF mRNA Analysis

**Figure 6:**
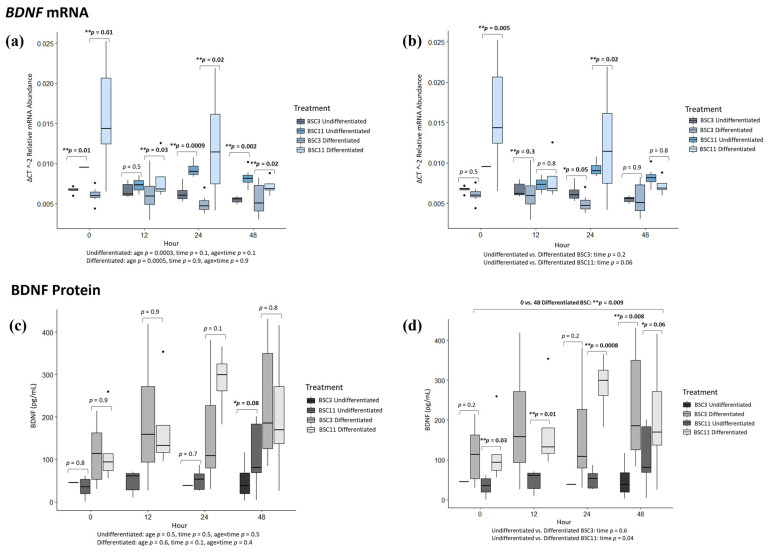
Average BDNF mRNA expression and protein secretion. The fixed effect results are listed at the bottom of each figure. (**a**) mRNA of undifferentiated and differentiated BSC3 vs. BSC11; (**b**) mRNA of undifferentiated vs. differentiated BSC3 and undifferentiated vs. differentiated BSC11; (**c**) proteins of undifferentiated and differentiated BSC3 vs. BSC11; (**d**) proteins of undifferentiated vs. differentiated BSC3 and undifferentiated vs. differentiated BSC11. The mRNA is in relative abundance and normalized to 18S ribosomal RNA. Whiskers represent 1.5 times the interquartile range, and * represents a trend; ** represents significance. Figures report the means of three repeated measures; BSC3 came from three-month-old steers (*n* = 3); and BSC11 came from 11-month-old steers (*n* = 3).

*BDNF* mRNA expression was challenging to detect, especially at 0 h in the undifferentiated BSC. Undifferentiated BSC3 vs. BSC11 had differences (*p* ≤ 0.01) at 0, 24, and 48 h, but no difference (*p* = 0.5) at 12 h (Figure 6a). For the hour with differences, the undifferentiated BSC11 had higher *BDNF* expression than the undifferentiated BSC3. The combined undifferentiated data had no differences (*p* ≥ 0.1) for 0 vs. 12 and 0 vs. 24 h, but there was a trend (*p* = 0.05) for 0 vs. 48 h (Figure 6a). *BDNF* mRNA trended to increase, especially for the undifferentiated BSC11. For fixed effects, there was a difference (*p* = 0.0003) for age but no difference (*p* ≥ 0.1) for time or age × time. While not much mRNA was detected for undifferentiated BSC11, what was detected was higher than the levels expressed by undifferentiated BSC3. Differentiated BSC3 vs. BSC11 had differences (*p* ≤ 0.01) for 0, 12, 24, and 48 h (Figure 6a). In the combined differentiated data, there were no differences (*p* ≥ 0.5) for 0 vs. 12, 0 vs. 24, and 0 vs. 48 h (Figure 6a). There was a difference (*p* = 0.0005) for the fixed effect of age, but no difference (*p* = 0.9) for time or age × time.

The undifferentiated vs. differentiated BSC3 revealed a difference (*p* = 0.05) at 24 h but no difference (*p* ≥ 0.3) at 0, 12, or 48 h (Figure 5b). The combined BSC3 data had no difference (*p* ≥ 0.2) for 0 vs. 12, 0 vs. 24, and 0 vs. 48 h (Figure 6b), or for the fixed effect of time. Undifferentiated vs. differentiated BSC11 had differences (*p* ≤ 0.02) for 0 and 24 h, but no difference (*p* ≥ 0.8) for 12 and 48 h (Figure 6b). The combined BSC11 had differences (*p* = 0.05) for the comparisons of 0 vs. 12 and 0 vs. 48 h, and no difference (*p* = 0.2) for 0 vs. 24 h (Figure 6b). The fixed effect of time trended towards a difference (*p* = 0.06), which is attributed to the decrease in *BDNF* expression over time.

### 3.9. BDNF Protein Analysis

The BDNF protein was difficult to detect in the culture medium for the undifferentiated BSC. Specifically, at 0, 12, and 24 h, little to no protein was detected from the undifferentiated BSC3 media samples, as many samples were out of range (undetectable) by the ELSA. This is still relevant data to report, though, as it could be physiologically relevant that the undifferentiated BSC3 did not secrete much protein in comparison to the other cells and treatments. Undifferentiated BSC3 vs. BSC11 had no differences (*p* ≥ 0.1) for 0, 12, and 24 h, and there was a trend (*p* = 0.08) at 48 h (Figure 6c). At 48 h, the undifferentiated BSC11 culture medium had higher BDNF protein than the undifferentiated BSC3 culture medium. The combined undifferentiated data also showed no differences (*p* ≥ 0.2) for 0 vs. 12 (no *p*-value), 0 vs. 24, and 0 vs. 48 h (Figure 6c), or for fixed effects of age, time, or age × time. Differentiated BSC3 vs. BSC11 had no differences (*p* ≥ 0.1) for 0, 12, 24, and 48 h (Figure 6c). The combined differentiated data had trends (*p* ≤ 0.1) for 0 vs. 24 and 0 vs. 48 h, but no differences (*p* ≥ 0.1) for 0 vs. 12 h (Figure 6c), or the fixed effects of age, time, or age × time.

Undifferentiated vs. differentiated BSC3 media samples had no difference (*p* = 0.2) for 0, 12 (no *p*-value), and 24 h (Figure 6d). There was a difference (*p* = 0.008) at 48 h. There was substantially more BDNF protein in the differentiated BSC3 media than the undifferentiated BSC3 media at the 48 h timepoint. The combined BSC3 data had no difference (*p* ≥ 0.3) for 0 vs. 12 (no *p*-value), 0 vs. 24, and 0 vs. 48 h (Figure 6d), or for the fixed effect of time. Undifferentiated BSC11 vs. differentiated BSC11 had differences (*p* ≤ 0.03) at 0, 12, and 24 h and a trend (*p* = 0.06) at 48 h (Figure 6d). The differentiated BSC11 media contained higher levels of BDNF than the undifferentiated BSC11 media samples. The combined BSC11 data showed no difference (*p* = 0.3) for 0 vs. 12 h; there was a trend (*p* = 0.07) for 0 vs. 24 h; and significance (*p* = 0.009) for 0 vs. 48 h (Figure 6d). At 24 and 48 h, differentiated BSC11 had higher levels of BDNF than the 0 h samples, and the undifferentiated BSC11 samples also appeared to have an increase in secretion. There was a difference (*p* = 0.04) for the fixed effect of time, as over time BDNF increased in the media.

The protein and mRNA results do not necessarily align for this target, but mRNA does not directly translate to protein, so essentially, protein and mRNA are not being evaluated at the exact same time point. The mRNA seen in the figure needs to be translated into a protein yet. The data support the finding that BDNF is a myokine produced by BSCs in vitro. From the results of the BDNF protein, it does appear that BDNF is associated with myogenic differentiation, as there was more protein in the media from cells induced into differentiation compared to cells that had not been differentiated.

## 4. Discussion

There is little information available on these myokines in livestock species. These select proteins have been heavily studied as exercise-induced myokines [46,47,48]. Cattle are not subjected to the same type of exercise in their natural environment as presented in human and biomedical exercise studies. Due to the lack of evidence, it is unknown if some of these proteins are myokines in cattle and if they differ from human and biomedical models. This is why this study was designed to evaluate if these are myokines derived from the primary muscle cells of cattle. Additionally, there is limited to no information on these proteins in the growth and development of cattle skeletal muscle, and it would be important to evaluate whether these proteins are associated with any meat quality characteristics in the future. Other studies in biomedical model research do indicate that IL-15, CTRP15, FNDC5, and BDNF are associated with muscle growth, metabolism, and/or repair [49,50,51,52,53,54], and it is possible that future studies of these myokines in cattle may identify similar effects on muscle in vivo. IL-6 has been shown to cause muscle atrophy with long-term high exposure but does have properties that still support muscle growth in humans and mice [55,56,57]. In the present study, primary cultures of BSCs were found to secrete IL-6, IL-15, CTRP15, and BDNF, which is suggestive of their potential role as myokines. Even though BSCs expressed *FNDC5*, we were unable to confirm the presence of the cognate protein, irisin. To the best of our knowledge, this is the first report of these myokines being expressed and secreted by bovine-derived skeletal muscle cells.

### 4.1. IL-6 and IL-15

IL-6 and IL-15 are both well-known cytokines [58,59] that also act as autocrine signaling myokines. IL-6 promotes human satellite cell proliferation [60] as well as differentiation and muscle cell hypertrophy in mice [28]. Both IL-6 and IL-15 are metabolic regulators and energy sensing myokines. To the authors’ knowledge, there are no other studies that have evaluated IL-6 and IL-15 in BSCs from two different age groups of cattle. Porcine work has identified that each of these myokines facilitates different roles in pigs and cells derived from pigs. In myostatin knock-out pigs, IL-6 was identified to promote fat browning [61]. When using porcine muscle satellite cells and adipocytes in co-culture, IL-15 appeared to promote satellite cell differentiation, and inhibit stromal vascular cell differentiation while being associated with a decrease in leptin secretion from adipocytes [62]. Others have suggested that the myokine IL-6 increases glucose uptake and metabolism in human skeletal muscle in both exercising and resting muscle cells [63,64]. When IL-15 is applied to C2C12 myoblasts, Jak3/STAT3 signaling increases, and the GLUT4 transporter is translocated to the cell surface, which increases glucose uptake [65]. In this study, *IL-6* expression was detected in both the undifferentiated and differentiated BSCs. There was an increase in expression over time where the differentiated BSCs had elevated expression levels. The transcript abundance of *IL-6* was also greater in BSCs harvested from 11-month-old steers than in 3-month-old steers. In previous literature, muscle cell proliferation and differentiation have been shown to influence IL-6 production [28,66], and in this work, it was identified that IL-6 −/− mice had defective proliferation. This is why it is not surprising to detect IL-6 protein in the undifferentiated BSCs, and possibly why we detected it at lower levels in the differentiated BSCs. Interestingly, the level of IL-6 protein detected in the differentiated BSCs did not match the mRNA. Although speculative, the difficulty in detecting protein in the differentiated BSCs could have been due to the differentiation media used, as this medium contained horse serum, whereas the IL-6 ELISA was bovine-specific. The presence of equine protein may have inhibited the effective detection of bovine IL-6. However, it should be mentioned that all ELISA assays were bovine-specific in this study, and we did not observe the same issue across the different targets. The degree of cross-reactivity of equine myokines in the media could vary between the proteins of interest. A study with C2C12 cells supports our findings, as it suggested that expression and secretion of IL-6 were enhanced when cells were induced into differentiation. This same study also utilized murine myoblasts that were *IL-6* −/− and identified that the lack of IL-6 impaired myoblast differentiation [66]. Another study highlighted that the concentration of IL-6 protein does matter for cell signaling, with a low concentration associated with proliferation and a high concentration associated with the induction of differentiation in both C2C12 myoblasts and human muscle satellite cells [67]. It could also be possible that IL-6 is acutely regulated in BSCs at the mRNA and protein levels, and our time points could have been inadequate to characterize these changes. This study determined that BSCs actively proliferating or maturing express and secrete IL-6.

As with *IL-6*, *IL-15* expression was higher in the differentiated BSCs, but interestingly, between 0 and 48 h, there was a decrease in expression in the undifferentiated BSCs. As seen in Figure 1, there was a fusion of cells even in the undifferentiated cultures at 48 h. Cell fusion is a sign of differentiation, which is likely a natural result of limited space and high confluency. Uninduced differentiation is consistent with primary cell culture, as a certain population of cells will begin to differentiate after 48 h of culture and beyond. We hypothesize that with differentiation occurring, there would be a corresponding increase in *IL-15* expression at 48 h. Additional time points would help identify if the natural differentiation of BSCs stimulates *IL-15* expression. There was broad variation in protein secretion, which did not differ across treatments or with time. It was not surprising to see that IL-15 expression was elevated, as IL-15 has been shown to stimulate differentiation in C2 cells when IGF-1 is inhibited [68], and research using human muscle cells proposed that IL-15 promotes myoblast fusion in developing primary myofibers [50]. Additional work with C2C12 cells observed that *IL-15* expression was upregulated in differentiated myotubes in comparison to undifferentiated myoblasts [69]. This relates to what we are seeing in this study, where we start to observe elevated IL-15 from cells induced into differentiation compared to undifferentiated cells. From these results, it appears that IL-15 could be important for differentiation, and it decreases slightly when fusion in the cells commences. Further work should be conducted to confirm its presence with bovine muscle cell fusion, as it was not thoroughly tested here.

### 4.2. CTRP15 and FNDC5

CTRP15 and FNDC5 have been classified as both adipokines and myokines as they are known regulators of adipose tissue and muscle [26,70,71]. To the best of our knowledge, CTRP15 has not been identified as being produced by BSCs, and little is known about its functions in the muscles of cattle. This study confirms its expression and secretion from BSCs. At 24 and 48 h, the undifferentiated BSCs express higher levels than the differentiated BSCs, regardless of the BSC age category. In addition, the expression of *CTRP15* in differentiated BSCs decreased between 0 and 48 h. Similarly, protein secretion was higher in the undifferentiated than the differentiated BSC, and between 0 and 12 h, protein secretion decreased in the undifferentiated BSC. Work by others suggests that *CTRP15* is highly expressed by murine and human skeletal muscle. This same work observed that differentiated C2C12 cells express more *CTRP15* than myoblasts [26]. Another study observed *CTRP15* expression in C2C12 myotubes [72]. This trend is opposite of the data of the current study, as proliferating cells were found to express and secrete more CTRP15 than differentiated cells. We hypothesize that this could be a species-related difference, but further evaluation is needed. The pathway and function of this myokine could be further evaluated in the proliferation and differentiation of many species, as there is limited information on this particular protein with regard to how it could influence cellular functions.

As covered in the introduction, there is *FNDC5* one study in cattle that has evaluated this gene and the irisin myokine [5]. Our findings with this target were that differentiated BSC3 had a higher relative mRNA abundance than undifferentiated BSC3 at every time point tested. The same relationship was observed for the BSC11 at 48 h. Additionally, there was an increase in *FNDC5* expression for the differentiated BSCs between 0 and 48 h. This would suggest that *FNDC5* could be involved in differentiation or be stimulated by induced differentiation. The mRNA does not directly translate to protein, which would signal differentiation. Studies using primary human skeletal muscle cells from obese but healthy patients identified increased *FNDC5* expression during myogenic differentiation [73], and human skeletal muscle myotubes express *FNDC5* and secrete irisin [74]. An in vitro study with L6 rat myoblasts observed that *FNDC5* expression increased during differentiation and actually peaked at days 5 and 7 of differentiation, suggesting there is a temporal pattern for *FNDC5* during differentiation [75]. These previous studies support the idea that *FNDC5* is important to the differentiation process. The results of this experiment suggest that *FNDC5* may be involved in the differentiation process of BSCs, which aligns with this previous work, but without protein quantification, this requires further assessment.

### 4.3. BDNF

Very little about BDNF is known in livestock species when it is secreted from the skeletal muscle. It is known that BDNF is important for neural development and adaptability to stress. Work in pigs observed that pigs living in enriched environments had higher BDNF in the serum compared to pigs living in a barren environment [76]. The BDNF levels in that study did not identify muscle as the tissue secreting the BDNF protein, but we expect that it could be contributing to the levels found in the serum. How animals handle stress does affect health and growth, which is why it is important to identify if BDNF is a myokine in livestock and, if so, at what levels. Our study established that this protein is a myokine produced by BSCs. There were difficulties detecting *BDNF* mRNA expression and protein secretion depending on time points and treatments in this study. At 0 h, expression was hardly detected for the undifferentiated BSC11, but given that it was well detected in each of the other groups, we report these data for cautious interpretation. As mentioned earlier, protein detection may have been influenced by the presence of differentiation media. The relative abundance of *BDNF* at 0 and 24 h for the differentiated BSC11 also had higher variation in the level detected than any of the other time points. From 0 to 24 h, there was little to no detection in the undifferentiated BSC3, but between 0 and 48 h, protein secretion in the differentiated BSCs did increase. At each time point, the differentiated BSC11 secreted more protein than the undifferentiated BSC11. A study using rat L6 skeletal muscle cells observed that *BDNF* was not expressed in mature muscle fibers. That study found that *BDNF* expression was repressed in differentiation and that reducing endogenous levels of BDNF promoted myogenic differentiation, and when there was expression, it was associated with cells expressing Pax3 and Pax7 [33]. Another study using *BDNF* knock-out mice identified that its expression was important for normal satellite cell function, but in contrast to the first study, the lack of BDNF impaired differentiation and regeneration [77]. Both studies identified that BDNF plays an important role in muscle satellite cell maintenance. Based on this previous work, we would expect that BSCs would express and secrete BDNF at our 0 h timepoint in this study, which was not the result observed. This current study confirms that BDNF is expressed and secreted by BSCs, but further work needs to be carried out to elucidate the function of BDNF in BSCs.

The results of this study are indicative of these proteins supporting muscle growth in cattle, which would align with their functions in other species, as mentioned previously. These myokines are also known to contribute to the regulation of energy in multiple tissue types, including adipose and skeletal muscle. Further, there is evidence of muscle adipose crosstalk for all five targets as outlined in reviews [78,79,80]. In human and biomedical models, these myokines are hypothesized to help modulate obesity by reducing fat deposition [81]. In cattle, a reduction in fat deposition is not ideal, especially in cattle intended for harvest. Intramuscular fat, also referred to as marbling, is very important for meat quality and is highly desirable [82]. This study evaluated the expression and secretion of these proteins by BSCs. Future work is needed to identify the functionality of these proteins in bovine muscle as well as whether they influence adipocyte development and intramuscular marbling in cattle. In this work, we observed the expression and secretion of IL-6, IL-15, CTRP15, and BDNF by bovine-derived muscle cells, but we were unable to accurately quantify the irisin/FNDC5 protein. Differences were identified for each protein detected, with observed differences in undifferentiated and differentiated BSC, temporal patterns in mRNA expression, as well as a few age-related differences in regard to the harvest age of BSCs.

## 5. Conclusions

To conclude, IL-6, IL-15, CTRP15, and BDNF are all expressed and secreted by a primary culture of BSCs, indicating that they are myokines. *FNDC5* mRNA was detectable, but protein secretion was not confirmed. This study proposes that IL-6, 1L-15, and FNDC5 are involved in cultured BSC differentiation, and CTRP15 may be more associated with BSC proliferation and is down-regulated during differentiation in BSCs. BDNF could be involved in differentiation, but this requires further analysis. The results from this study suggest that these targets could be involved in primary muscle cell growth in cattle.

## Figures and Tables

**Table 1:** Averages of each myokine detected in the raw unconditioned media. Both types of growth media that had never been exposed to cells were assessed to identify whether the myokines of interest were naturally present in the media.

Myokine	Undifferentiated Media Average (pg/mL)	Differentiated Media Average (pg/mL)
IL-6	0.697	Undetectable
IL-15	24	30
CTRP15	737	731
BDNF	Undetectable	Undetectable

**Table 2:** Custom TaqMan primer and probes. Labels: FP = forward primer; RP = reverse primer.

Target	Primers/Probes	Probe Type	Probe Dye
*18S*	FP:CCACGCGAGATTGAGCAAT RP:GCAGCCCCGGACATCTAA Probe: ACAGGTCTGTGATGCC	MGB	FAM
*IL-6*	FP: GCCCTCCAGGAACAGCTATG RP:GCCCTCCAGGAACAGCTATG Probe: ACTCCCGCTTCACAAG	MGB	VIC
*IL-15*	FP: TCATGTCTTCATTTTGGGCTGTA RP:CATACTGCCAGTTTGCTTCTGTTT Probe: AGTGCAAGTCTTCC	MGB	FAM
*CTRP15*	FP: CGGTCAACGGCGTTCTCTAT RP: CACTGGCATTGTCCAAGAAGAC Probe: TGCAGGCCGGGCAGTACACCTC	QSY	ABY
*FNDC5*	FP: TCGGCCCCAGTGAACGT RP: CCAAGACATCCCAGCTTACCA Probe: CGTCAGGCACCTCAAGGCCAACTC	MGB	FAM
*BDNF*	FP: GAAAACAATAAAGACGCGGACAT RP: GGGCTCCAAAGGCACTTGA Probe: TACACGTCCCGGGTGATGCTCAGC	ABY	QSY

## Data Availability

The data presented in this study are available upon request from the corresponding author. The data are not publicly available due to restrictions put in place by the funding agency.
